# Cardiovascular Comorbidities in Chronic Obstructive Pulmonary Disease (COPD)—Current Considerations for Clinical Practice

**DOI:** 10.3390/jcm8010069

**Published:** 2019-01-10

**Authors:** Frederik Trinkmann, Joachim Saur, Martin Borggrefe, Ibrahim Akin

**Affiliations:** 1 1st Department of Medicine (Cardiology, Angiology, Pulmonary and Intensive Care), University Medical Center Mannheim, Medical Faculty Mannheim, Heidelberg University, 68167 Mannheim, Germany; jsaur@uni-mannheim.de (J.S.); martin.borggrefe@umm.de (M.B.); ibrahim.akin@umm.de (I.A.); 2 DZHK (German Center for Cardiovascular Research), partner site Mannheim, University Medical Center Mannheim, Medical Faculty Mannheim, Heidelberg University, 68167 Mannheim, Germany

**Keywords:** COPD, comorbidities, cardiovascular, diagnostics, therapy

## Abstract

In patients with chronic obstructive pulmonary disease (COPD), cardiovascular comorbidities are highly prevalent and associated with considerable morbidity and mortality. This coincidence is increasingly seen in context of a “cardiopulmonary continuum” rather than being simply attributed to shared risk factors such as cigarette smoking. Overlapping symptoms such as dyspnea or chest pain lead to a worse prognosis due to missed concomitant diagnoses. Moreover, medication is often withheld as a result of unfounded concerns about side effects. Despite the frequent coincidence, current guidelines are still mostly restricted to the management of the individual disease. Future diagnostic and therapeutic strategies should therefore be guided by an integrative perspective as well as a refined phenotyping of disease entities.

## 1. Introduction

**Figure 1:**
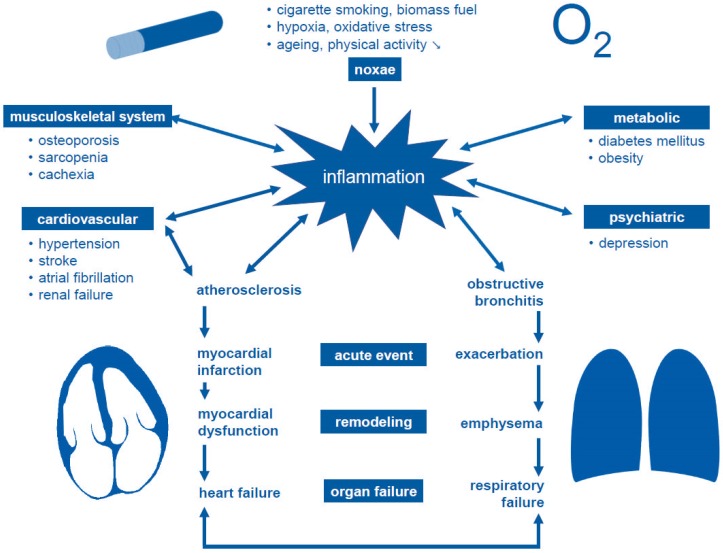
Cardiovascular and pulmonary disease in the context of inflammation (“cardiopulmonary continuum”, modified after [1,4]).

Concomitant cardiovascular disease in chronic obstructive pulmonary disease (COPD) is increasingly seen in context of a “cardiopulmonary continuum” [1] rather than being simply attributed to shared risk factors such as cigarette smoking. In recent years, complex cardio-respiratory interactions were identified. These are not restricted to structural, vascular, and genetic factors. Both disease entities are centrally linked to systemic inflammation (Figure 1). Various factors contribute to this process, most notably inhaled noxae but also hypoxia, oxidative stress, ageing, and reduced physical activity. Inflammatory levels are further increased by disorders which themselves lead to aggravation or development of comorbidities. These include atherosclerosis and obstructive bronchitis, being the basis for acute events such as myocardial infarction or acute exacerbations. Structural changes during remodeling eventually lead to organ failure which clinically presents as heart failure or respiratory failure, respectively.

Different clinical phenotypes of COPD may be associated with specific inflammatory signaling pathways. While cardio-metabolic disease frequently leads to airway-predominant COPD, sarcopenia, and osteoporosis is predominantly found in patients with emphysema. In four out of five COPD patients, at least one comorbidity is present, with cardiovascular entities being most frequent [2,3]. This multimorbidity is a major challenge to health care systems world-wide, requiring an integrative rather than highly specialized approach.

## 2. Cardiovascular Risk

Patients with COPD have a two- to three-fold increased cardiovascular morbidity and mortality [5]. It is associated with disease severity [6], and inflammation is seen to be particularly important. This finds its expression in elevated markers of inflammation even in stable pulmonary disease [7], indicating a link between local pulmonary and systemic inflammatory processes. Hence, therapeutic interventions based on inhaled corticosteroids (ICS) modulating systemic inflammation seem attractive. Being a cornerstone of anti-inflammatory therapy in bronchial asthma, their use is restricted to distinct phenotypes in COPD. Main indications include patients with frequent exacerbations or blood eosinophilia according to current Global Initiative for Chronic Obstructive Lung Disease (GOLD) recommendations [8]. Presence of eosinophilic inflammation can be assessed using fractional exhaled nitric oxide (FeNO). Recently, it was shown that patients with high FeNO levels and non-specific respiratory symptoms were more likely to respond to ICS as compared to placebo [9]. However, no differences in cardiovascular events were found between patients at risk treated with either fluticasone furoate, vilanterol, or their combination as compared to placebo [10]. Although therapy did not alter plasma cardiac troponin I levels, the latter were recently shown to predict future cardiovascular events [11]. In contrast, studies targeting cardiovascular safety of oral phosphodiesterase inhibitor roflumilast revealed a 35% reduction of cardiovascular events [12]. Likewise, a decrease in hyperinflation was associated with a reduction of systemic inflammation. This may explain the positive effect of dual bronchodilation as compared to long-acting beta agonist (LABA)/ICS combination therapy that are still used frequently [13].

Statins are primarily used for reduction of serum cholesterol levels. Apart from this, pleiotropic immune modulatory as well as anti-inflammatory effects have been described [14,15]. These may independently contribute to a decrease of airway inflammation in theory. A 38% reduction of overall mortality and 31% reduction of myocardial infarction was previously found in a systematic review of observational research [16]. Although there seems to be a correlation between reduction of forced expiratory volume in one second (FEV_1_) and cardiovascular mortality [17], statin treatment is neither associated with improved pulmonary nor vascular function in general [18]. Evidence from observational studies towards a lower exacerbation rate could not be consistently confirmed in controlled trials [19]. However, there was a positive effect in a prespecified subgroup of patients with supra-median inflammatory levels [18]. A decrease in systemic inflammation markers was seen in 79% of patients receiving pravastatin in a randomized trial and associated with improvement in exercise time as compared to placebo [20]. Statin doses have been a major point of criticism. Patients with high inflammatory levels particularly seem to benefit most from therapy [21]. Although being potentially attractive, these contradicting study results suggest that the concept does not completely capture the complex disease interactions. Therefore, improved phenotyping of COPD may be a starting point for innovative protocols of urgently needed controlled trials. 

Problematically, current risk stratification scores do not sufficiently include the increased cardiovascular risk associated with COPD [22]. In contrast to primary prevention, secondary prevention statin therapy is associated with a decrease in cardiovascular events and mortality [23]. However, this patient group is neither addressed by the target value approaches recommended in the 2016 European Society of Cardiology (ESC)/European Atherosclerosis Society (EAS) [24] and 2017 American Association of Clinical Endocrinologists (AACE)/American College of Endocrinology (ACE) guidelines [25] nor by the statin benefit group approach that is still currently pursued by 2013 American College of Cardiology (ACC)/American Heart Association (AHA) guideline [26]. COPD itself is increasingly recognized as an independent cardiovascular risk factor, in addition to coronary heart disease, peripheral arterial occlusive disease, diabetes mellitus, or renal failure [27,28]. Hence, statin therapy may be justified in patients with COPD for secondary prevention [29]. However, it should be noted that randomized controlled trials are lacking. Modification of the cardiovascular risk profile outside current guideline recommendations is a challenging task. It requires individual and informed consent between patients and physicians.

## 3. Coronary Heart Disease

Endothelial dysfunction fundamentally contributes to the development of atherosclerosis that finally leads to coronary heart disease (CHD). The process is further accelerated by systemic inflammation and oxidative stress. As a result, approximately one out of six COPD patients suffers from concomitant CHD [30,31]. Moreover, COPD exacerbations are associated with a transient deterioration of endothelial function. This translates into an increased risk for macrovascular complications such as myocardial infarction and stroke. Additionally, subsequent loss of lung function is associated with a long-term increase in arterial stiffness [32]. In the acute setting, all-cause mortality can be predicted by elevated levels of cardiac troponin [33]. Coronary artery calcification correlates with dyspnea, exercise capacity and all-cause mortality. This indicates a link between coronary heart disease and poor clinical outcome in patients with COPD [34]. Therefore, identification of patients with a high coronary artery calcium score is important in order to provide appropriate targeted therapy and cardiovascular risk modification. Current guidelines are mostly restricted to the individual cardiac or respiratory disease [8,35]. Nevertheless, an integrative perspective is warranted, especially as long-term data of patients with COPD and CHD is scarce. Smoking cessation still is the most important measure for secondary prevention and will remain so in the foreseeable future. Concomitant COPD is not diagnosed in around 80% of patients undergoing coronary intervention [36]. This mostly affects early or moderate stages in which better preventive and therapeutic options would still be available. Vice versa, electrocardiographic signs of previous myocardial infraction are not recognized in 70% of patients presenting with acute COPD exacerbations [37]. Clinical assessment is impeded by frequently present atypical angina pectoris, dyspnea or palpitations leading to misinterpretation [4].

Beta blockers are a corner stone of drug treatment in stable CHD while being associated with symptom control and improved prognosis. Nevertheless, they are often withheld or under-dosed in COPD patients. This is a result of unfounded concerns about side effects such as aggravation of dyspnea or bronchoconstriction [38,39] and associated with worse prognosis after myocardial infarction. Cardio-selective beta blockers were shown to be safe when (partly)-reversible obstruction is present [40]. Retrospective analyses even suggest a reduced mortality and exacerbation frequency in this setting [41]. Ivabradine is a reasonable substitute in patients with sinus rhythm intolerant to beta blockers also improving diagnosis. Calcium channel blockers and nitrates are alternatives in patients with angina pectoris [42]. Medication inhibiting the renin–angiotensin–aldosterone system (RAAS) are indicated in patients with heart failure, arterial hypertension or diabetes mellitus and reduce morbidity as well as mortality [35]. Additionally, positive effects were postulated on systemic inflammation, skeletal muscle function, peripheral oxygen consumption, and erythrocytopoiesis [43]. Although these could not be fully confirmed in interventional trials [44], potential benefits should be interpreted in the context of reducing cardiovascular mortality and warrant further investigation. Moreover, no evidence for an increased incidence of bronchoconstriction or cough exists [45,46]. While acetylsalicylic acid is primarily associated with an increased exacerbation risk in asthma, reversible P2Y₁₂-antagonists (ticagrelor, cangrelor) are suspected to cause dyspnea. In contrast, irreversible substances such as clopidogrel and prasugrel do not seem to be associated with increased respiratory symptoms [47].

Still, there is no evidence that would justify deviation from current therapeutic recommendations in patients with concomitant COPD and CHD [8]. Concerns about the cardiovascular safety of beta mimetic or antimuscarinic drugs should always be evaluated in context of an inherently increased cardiovascular risk in COPD. During initiation of LABAs and anticholinergics, an increased risk of cardiovascular events was found in a retrospective cohort study in the elderly [48]. Moreover, betaagonists may precipitate ischemia, congestive heart failure, arrhythmias, and sudden death [49]. Apart from the beforementioned SUMMIT study, meta-analyses even suggest a reduction of severe cardiovascular events by tiotropium bromide [50]. Likewise, it could be demonstrated that long-acting muscarinic antagonists were associated with a reduced risk of acute myocardial infarction when applied via a dry powder inhaler. Beta agonists alone were associated with an increased risk of acute myocardial infarction while combination with ICS was not [51]. In general, long-acting substances have an acceptable safety profile [52]. However, it seems reasonable to avoid high doses of short-acting drugs during acute coronary syndrome (Table 1).

Myocardial revascularization is associated with worse long-term results in patients with concomitant COPD [53,54]. This may be the clinical expression of rather diffuse lesions [55]. These are harder to tackle using both surgery (coronary arterial bypass graft, CABG) as well as interventional (percutaneous coronary intervention, PCI) approaches. Moreover, COPD patients are less likely to receive guideline-based medical therapy. This may further contribute to the increased mortality as well as revascularization rates. When choosing between CABG vs. PCI as the optimal approach, perioperative risk (sternotomy, possibly extracorporeal circulation) has to be carefully evaluated in context of higher rates of restenosis and the lack of data showing an improved prognosis. Risk–benefit assessment should be performed in interdisciplinary Heart Teams guided by prediction scores such as SYNTAX II or EuroSCORE II. Both include COPD as independent risk factors [56].

## 4. Heart Failure

Left heart failure is diagnosed first-time in one out of five COPD patients after extensive cardiovascular work-up [64]. Vice versa, one out of three heart failure patients suffers from obstructive ventilation disorders [65]. These frequent coincidences are not only associated with diagnostic difficulties, but also with worse prognosis (five-year survival 31% vs. 71%) [66]. Concomitant obstructive ventilation disorders during acute left heart failure are fully reversible in half of the cases after six months. However, initial hyperinflation has a predictive value for diagnosis of COPD [67]. Elevated brain natriuretic peptide concentrations (BNP) above 500 pg/mL are more likely to be caused by left heart failure in acute settings [68]. In contrast, pulmonary differential diagnoses are more frequently found if levels are below 100 pg/mL. Between these thresholds, diagnostic performance for detection of biventricular heart failure and right heart failure alone is limited. In general, the latter tends to lower BNP values [69]. Evaluation of patient history as well as clinical signs can be valuable in differential diagnosis. For example, acute exacerbations of COPD often lead to apical and ventral wheezing. In contrast, alveolar edema due to left heart decompensation leads to fine crackles but also wheezing of lower parts of the lung. Moreover, interpretation of conventional X-ray is complicated by chronic structural changes. At the same time, they can superimpose or mimic pulmonary venous congestion. Hence, evaluation of pulmonary and cardiac limitations should only be done in stable, euvolemic phases of about three months’ time frame. Although no robust data is available evaluating the efficacy of screening programs, both organ systems seem worth to be included in diagnostic considerations. This holds especially as non-invasive diagnostic tools as well as treatment strategies are widely available. In stable COPD, transthoracic echocardiography should be initially performed. However, poor acoustic window may be present in up to 50% the patients depending on the degree of airflow limitation [70]. Evaluation of heart failure with preserved ejection fraction (HFpEF) is particularly challenging in this setting and associated with false diagnoses due to pulmonary comorbidity [71]. Alternatives include cardiac magnetic resonance imaging as well as cardio pulmonary exercise testing. The latter additionally allows functional phenotyping of the disease [72]. Natriuretic peptides are primarily used for excluding heart failure at thresholds below 125 pg/mL for NT-pro-BNP and 35 pg/mL for BNP, respectively [73]. Elevated values should be further evaluated.

There is currently no evidence to pursue treatment strategies differing from current guidelines for COPD with concomitant heart failure. However, beta blockers are still frequently withheld in this setting although they were shown to considerably reduce mortality [74]. Cardio selective drugs should be preferred if possible [75]. As compared to unselective substances such as carvedilol, bisoprolol was shown to cause fewer side effects and even improve lung function [76]. The value of novel therapeutic options such as angiotensin receptor–neprilysin inhibition (sacubitril/valsartan) in patients with heart failure and concomitant COPD warrants further evaluation. Recently, dual bronchodilator therapy was demonstrated to improve cardiac function. It may therefore warrant early medical treatment in COPD patients with signs of hyperinflation [77]. Table 1 summarizes indications and possible pitfalls of frequently used medication in patients with COPD and cardiovascular disease, respectively.

## 5. Arterial Hypertension

Arterial hypertension is comparably frequent in COPD patients and the general population. Prevalence is about 50% and increasing with age [78]. Nevertheless, the overall elevated cardiovascular risk in COPD may be a product of this common condition and potentiating effects of other risk factors such as diabetes mellitus and cigarette smoking. Moreover, higher central blood pressure values and arterial stiffness are also found indicating premature atherosclerosis [79]. Central blood pressure values are more strongly correlated with markers of hypertensive end-organ damage in general [80,81,82] and better predict cardiovascular outcomes [81,83]. Moreover, important differences between classes of antihypertensive drugs were found regarding their effect on central blood pressure in general populations [84,85], despite having a similar impact on brachial values [86,87]. However, there is currently no evidence for application of alternative blood pressure target values or medication when arterial hypertension is present in COPD patients. Theoretical advantages of calcium antagonists due to smooth muscle relaxation only translate into little clinical effect [88]. In recent years, several techniques for non-invasive determination of central blood pressure became available [89,90,91]. They have the potential to facilitate application in clinical routine. However, it remains to be determined whether measurement of central blood pressure will also improve differential antihypertensive therapy in patients with COPD.

## 6. Pulmonary Hypertension

Increased pulmonary blood pressure can be most commonly attributed to left heart disease (Nizza group 2) or pulmonary disease (Nizza group 3). In the latter, development of pulmonary hypertension is associated with a worse prognosis than in patients with COPD alone. Interestingly, there is a morphological overlap between lesions found in group 1 (pulmonary arterial hypertension, PAH) and group 3 pulmonary hypertension, respectively. The five-year survival rate (36%) is primarily driven by hemodynamic and not ventilatory parameters [92]. Likewise, a two-fold increase in mortality is found in patients with left heart disease (group 2). This is due to consecutive right heart failure and to date convincing therapeutic concepts are missing. When mitral valve disease is present additionally, surgical and interventional options should be evaluated as they may lead to considerable reduction in pulmonary arterial pressure [93]. In both groups 2 and 3, therapeutic response to targeted therapy as used in PAH (group 1) seems to be closely related to hemodynamic impairment. Identification of these subgroups requires a profound diagnostic work-up and should ideally be performed in expert centers following urgently required study protocols [92].

## 7. Cardiac Arrhythmias

Atrial fibrillation is common during acute COPD exacerbations and may complicate differential diagnosis [94]. Overall, there is an 1.8-fold increase in prevalence in COPD patients that is associated with the degree of lung function impairment [95]. Elevated pulmonary arterial pressures with consecutive right heart failure are made responsible. This is a result of hypoxemia, hypercapnia, and systemic inflammation, but also drug side effects. When initiating oral steroids or using high doses (≥7.5 mg prednisolone or equivalent), a 3.4-fold increase in risk for newly onset atrial fibrillation was seen [58]. In contrast, ICS was not associated with a higher risk [57]. In contrast to short-time use of theophylline, beta agonists were not associated with atrial fibrillation in this large case–control study (Table 1). Compensation of hypoxemia and respiratory acidosis are cornerstones of therapy. Both conditions were shown to negatively influence the effectivity of medication as well as electrical cardioversion [96]. Vice versa, the presence of COPD itself is a risk factor for unsuccessful catheter ablation therapy [97] as well as for recurrent atrial fibrillation after electrical cardioversion [98]. This may be due to anatomical changes in pulmonary circulation which are associated with right atrial foci in COPD patients [99]. Being less susceptible to electrical cardioversion, secondary multifocal atrial tachycardias have to be clearly differentiated from atrial fibrillation [100]. Apart from optimizing COPD therapy, verapamil or beta blockers are specific therapeutic options. Given the tremendous arrhythmogenic potential and availability of effective inhaled drugs, it should be possible to avoid theophylline effortlessly. However, caution may be warranted in short-acting bronchodilators. In contrast, benefits most often exceed the potential risks of long-acting substances. Non-dihydropyridine calcium channel blockers and cardio-selective beta blockers are considered to be safe for frequency control in COPD patients [40]. In contrast, class IA and IC antiarrhythmic drugs are often contraindicated due to concomitant CHD.

The overall elevated risk for sudden cardiac death further increases after five and a half years from initial diagnosis as well as in patients with frequent exacerbations [101]. Most often, ventricular arrhythmias are made responsible. Likewise, short-acting beta agonists, theophylline and oral steroids have arrhythmogenic potential due to a decrease in potassium concentrations [49,57]. Previous findings suggest that patients with COPD more frequently die during night time. This is potentially due to the reduced ventilation resulting in more ventricular ectopic episodes while they are asleep. Hence, therapeutic options in current guidelines include medication (beta blockers, amiodarone), implantable cardioverter defibrillator (ICD), and catheter ablation, but also cautious handling of QT-prolongating drugs [102]. Pulmonary toxicity, especially development of pulmonary fibrosis should be taken into consideration for long term amiodarone treatment. Incidence is dose dependent and estimated as high as 15%. Typical daily doses below 200 mg are associated with a 0.1 to 0.5% risk [63]. Therefore, symptoms as well as transfer factor should be monitored routinely. Sotalol is a class III antiarrhythmic alternative. However, it has the potential to cause bronchoconstriction due to its non-selective binding to both β1- and β2-adrenergic receptors.

## 8. Summary

Concomitant cardiovascular disease is frequent in COPD patients and associated with considerable morbidity and mortality. In recent years, complex cardio-respiratory interactions were identified. Rather than being simply attributed to shared risk factors such as cigarette smoking, coincident cardiovascular disease and COPD is increasingly seen in context of a “cardiopulmonary continuum”. Both entities are centrally linked to systemic inflammation. Current guidelines are still mostly restricted to the management of the individual disease. However, overlapping symptoms such as dyspnea or chest pain lead to a worse prognosis due to missed concomitant cardiac or pulmonary diagnoses. Their presence should hence lead to further evaluation using lung function testing, echocardiography and electrocardiography. Moreover, medication is often withheld due to unfounded concerns about side effects. Cardio-selective betablockers and long-acting bronchodilators both show an acceptable safety profile. In contrast, theophylline as well as high doses of short-acting bronchodilators and oral corticosteroids contain relevant proarrhythmic potential. Although drug therapy using steroids or statins seems to be attractive for influencing systemic inflammation, the concept does not fully capture the complex interactions. Various additional factors such as inhaled noxae, hypoxia, oxidative stress, ageing, and reduced physical activity contribute to this process. Different clinical phenotypes of COPD may be associated with specific inflammatory signaling pathways. Cardio-metabolic disease frequently leads to airway-predominant COPD. In contrast, sarcopenia and osteoporosis is predominantly found in patients with emphysema. Therefore, future diagnostic and therapeutic strategies should be guided by an integrative perspective as well as a refined phenotyping of the disease entities.

## Figures and Tables

**Table 1:** Frequently used medication in COPD and cardiovascular disease.

	Medication		Indication	Comment	References
**COPD**	steroids	inhaled	long-term therapy ^#^	good safety profile	[57]
		systemic	exacerbation	pro-arrhythmic potential	[58]
	beta agonists	short acting	exacerbation long-term therapy	pro-arrhythmic potential (high doses)	[50,51,52,59]
		long acting	long-term therapy	acceptable safety profile	
	muscarinic antagonists	short acting	exacerbation long-term therapy	pro-arrhythmic potential (high doses)	[50,52,60]
		long acting	long-term therapy	acceptable safety profile	
	PDE inhibitors	Roflumilast	long-term therapy	reduction of cardiovascular events	[12]
		Theophylline	(long-term therapy)	narrow therapeutic range and considerable pro-arrhythmic potential	[57,61]
**Cardiovascular Disease**	beta blockers	selective	heart failure, CHD, ACS, AHT, SVT, VT	often withheld or under dosed, prefer selective substances, overall good safety profile	[40,41]
		non-selective			
	antiplatelet therapy		CHD, ACS	dyspnea caused by reversible P2Y₁₂-antagonists (Ticagrelor, Cangrelor)	[47]
	Ivabradine		CHD, heart failure	alternative to beta blockers for anti-anginal and frequency control (sinus rhythm only)	[42,62]
	statins		CHD, dyslipidemia	secondary prevention, pleiotropic effects of immune system and inflammation	[14,15]
	ACE inhibitors		AHT, heart failure	no bronchoconstriction	[45,46]
	angiotensin receptor blockers			alternative (ACE inhibitor induced cough)	
	calcium channel blockers		AHT, CHD, SVT	alternative to beta blockers for anti-anginal and frequency control, smooth muscle relaxation (small clinical effect)	[42]
	nitrates		CHD	alternative to beta blockers for anti-anginal control	[42]
	amiodarone		SVT, VT	pulmonary toxicity	[63]

COPD: chronic obstructive pulmonary disease, PDE: phosphodiesterase, ACS: acute coronary syndrome, AHT: arterial hypertension, SVT: supraventricular tachyarrhythmia, VT: ventricular supraventricular, CHD: coronary heart disease, ACE: angiotensin converting enzyme. ^#^ in selected patients with frequent exacerbations or blood eosinophilia (see text for details).

## References

[1] Ukena C., Mahfoud F., Kindermann M., Kindermann I., Bals R., Voors A.A., van Veldhuisen D.J., Bohm M. (2010). The cardiopulmonary continuum systemic inflammation as ‘common soil’ of heart and lung disease. Int. J. Cardiol..

[2] Brown J.P., Martinez C.H. (2016). Chronic obstructive pulmonary disease comorbidities. Curr. Opin. Pulm. Med..

[3] Camiciottoli G., Bigazzi F., Magni C., Bonti V., Diciotti S., Bartolucci M., Mascalchi M., Pistolesi M. (2016). Prevalence of comorbidities according to predominant phenotype and severity of chronic obstructive pulmonary disease. Int. J. Chronic Obstruct. Pulm. Dis..

[4] Boschetto P., Beghe B., Fabbri L.M., Ceconi C. (2012). Link between chronic obstructive pulmonary disease and coronary artery disease: Implication for clinical practice. Respirology.

[5] Huiart L., Ernst P., Suissa S. (2005). Cardiovascular morbidity and mortality in COPD. Chest.

[6] Curkendall S.M., Lanes S., de Luise C., Stang M.R., Jones J.K., She D., Goehring E. (2006). Chronic obstructive pulmonary disease severity and cardiovascular outcomes. Eur. J. Epidemiol..

[7] Gan W.Q., Man S.F., Senthilselvan A., Sin D.D. (2004). Association between chronic obstructive pulmonary disease and systemic inflammation: A systematic review and a meta-analysis. Thorax.

[8] (2019). Global Strategy for the Diagnosis, Management and Prevention of COPD, Global Initiative for Chronic Obstructive Lung Disease (GOLD). https://goldcopd.org.

[9] Price D.B., Buhl R., Chan A., Freeman D., Gardener E., Godley C., Gruffydd-Jones K., McGarvey L., Ohta K., Ryan D. (2018). Fractional exhaled nitric oxide as a predictor of response to inhaled corticosteroids in patients with non-specific respiratory symptoms and insignificant bronchodilator reversibility: A randomised controlled trial. Lancet Respir. Med..

[10] Vestbo J., Anderson J.A., Brook R.D., Calverley P.M., Celli B.R., Crim C., Martinez F., Yates J., Newby D.E., Investigators S. (2016). Fluticasone furoate and vilanterol and survival in chronic obstructive pulmonary disease with heightened cardiovascular risk (SUMMIT): A double-blind randomised controlled trial. Lancet.

[11] Adamson P.D., Anderson J.A., Brook R.D., Calverley P.M.A., Celli B.R., Cowans N.J., Crim C., Dixon I.J., Martinez F.J., Newby D.E. (2018). Cardiac Troponin I and Cardiovascular Risk in Patients with Chronic Obstructive Pulmonary Disease. J. Am. Coll. Cardiol..

[12] White W.B., Cooke G.E., Kowey P.R., Calverley P.M., Bredenbroker D., Goehring U.M., Zhu H., Lakkis H., Mosberg H., Rowe P. (2013). Cardiovascular safety in patients receiving roflumilast for the treatment of COPD. Chest.

[13] Wedzicha J.A., Banerji D., Chapman K.R., Vestbo J., Roche N., Ayers R.T., Thach C., Fogel R., Patalano F., Vogelmeier C.F. (2016). Indacaterol-Glycopyrronium versus Salmeterol-Fluticasone for COPD. N. Engl. J. Med..

[14] Liao J.K., Laufs U. (2005). Pleiotropic effects of statins. Annu. Rev. Pharmacol. Toxicol..

[15] Oesterle A., Laufs U., Liao J.K. (2017). Pleiotropic Effects of Statins on the Cardiovascular System. Circ. Res..

[16] Cao C., Wu Y., Xu Z., Lv D., Zhang C., Lai T., Li W., Shen H. (2015). The effect of statins on chronic obstructive pulmonary disease exacerbation and mortality: A systematic review and meta-analysis of observational research. Sci. Rep..

[17] Anthonisen N.R., Connett J.E., Enright P.L., Manfreda J. (2002). Lung Health Study Research Group. Hospitalizations and mortality in the Lung Health Study. Am. J. Respir. Crit. Care Med..

[18] Neukamm A., Hoiseth A.D., Einvik G., Lehmann S., Hagve T.A., Soyseth V., Omland T. (2015). Rosuvastatin treatment in stable chronic obstructive pulmonary disease (RODEO): A randomized controlled trial. J. Intern. Med..

[19] Carlson A.A., Smith E.A., Reid D.J. (2015). The stats are in: An update on statin use in COPD. Int. J. Chronic Obstruct. Pulm. Dis..

[20] Lee T.M., Lin M.S., Chang N.C. (2008). Usefulness of C-reactive protein and interleukin-6 as predictors of outcomes in patients with chronic obstructive pulmonary disease receiving pravastatin. Am. J. Cardiol..

[21] Lahousse L., Loth D.W., Joos G.F., Hofman A., Leufkens H.G., Brusselle G.G., Stricker B.H. (2013). Statins, systemic inflammation and risk of death in COPD: The Rotterdam study. Pulm. Pharmacol. Ther..

[22] John M.E., Hussain S., Haddad M.A., Bolton C.E. (2014). M138 Do Standard Cardiovascular Risk Scores Identify Risk in Patients with Copd?. Thorax.

[23] Sheng X., Murphy M.J., MacDonald T.M., Schembri S., Simpson W., Winter J., Winter J.H., Wei L. (2012). Effect of statins on total cholesterol concentrations, cardiovascular morbidity, and all-cause mortality in chronic obstructive pulmonary disease: A population-based cohort study. Clin. Ther..

[24] Catapano A.L., Graham I., De Backer G., Wiklund O., Chapman M.J., Drexel H., Hoes A.W., Jennings C.S., Landmesser U., Pedersen T.R. (2016). 2016 ESC/EAS Guidelines for the Management of Dyslipidaemias. Eur. Heart J..

[25] Jellinger P.S., Handelsman Y., Rosenblit P.D., Bloomgarden Z.T., Fonseca V.A., Garber A.J., Grunberger G., Guerin C.K., Bell D.S.H., Mechanick J.I. (2017). American Association of Clinical Endocrinologists and American College of Endocrinology Guidelines for management of dyslipidemia and prevention of cardiovascular disease—Executive summary. Complete Appendix to Guidelines. Endocr. Pract..

[26] Stone N.J., Robinson J.G., Lichtenstein A.H., Bairey Merz C.N., Blum C.B., Eckel R.H., Goldberg A.C., Gordon D., Levy D., Lloyd-Jones D.M. (2014). 2013 ACC/AHA guideline on the treatment of blood cholesterol to reduce atherosclerotic cardiovascular risk in adults: A report of the American College of Cardiology/American Heart Association Task Force on Practice Guidelines. Circulation.

[27] De Lucas-Ramos P., Izquierdo-Alonso J.L., Rodriguez-Gonzalez Moro J.M., Frances J.F., Lozano P.V., Bellon-Cano J.M. (2012). Chronic obstructive pulmonary disease as a cardiovascular risk factor. Results of a case-control study (CONSISTE study). Int. J. Chronic Obstruct. Pulm. Dis..

[28] Durheim M.T., Cyr D.D., Lopes R.D., Thomas L.E., Tsuang W.M., Gersh B.J., Held C., Wallentin L., Granger C.B., Palmer S.M. (2016). Chronic obstructive pulmonary disease in patients with atrial fibrillation: Insights from the ARISTOTLE trial. Int. J. Cardiol..

[29] Andrade J., Ignaszewski A. (2008). Cardiovascular risk assessment: Identification of individuals at increased risk. BCMJ.

[30] Karch A., Vogelmeier C., Welte T., Bals R., Kauczor H.U., Biederer J., Heinrich J., Schulz H., Glaser S., Holle R. (2016). The German COPD cohort COSYCONET: Aims, methods and descriptive analysis of the study population at baseline. Respir. Med..

[31] Putcha N., Han M.K., Martinez C.H., Foreman M.G., Anzueto A.R., Casaburi R., Cho M.H., Hanania N.A., Hersh C.P., Kinney G.L. (2014). Comorbidities of COPD have a major impact on clinical outcomes, particularly in African Americans. Chronic Obstr. Pulm. Dis..

[32] McAllister D.A., Maclay J.D., Mills N.L., Mair G., Miller J., Anderson D., Newby D.E., Murchison J.T., Macnee W. (2007). Arterial stiffness is independently associated with emphysema severity in patients with chronic obstructive pulmonary disease. Am. J. Respir. Crit. Care Med..

[33] Pavasini R., d’Ascenzo F., Campo G., Biscaglia S., Ferri A., Contoli M., Papi A., Ceconi C., Ferrari R. (2015). Cardiac troponin elevation predicts all-cause mortality in patients with acute exacerbation of chronic obstructive pulmonary disease: Systematic review and meta-analysis. Int. J. Cardiol..

[34] Williams M.C., Murchison J.T., Edwards L.D., Agusti A., Bakke P., Calverley P.M., Celli B., Coxson H.O., Crim C., Lomas D.A. (2014). Coronary artery calcification is increased in patients with COPD and associated with increased morbidity and mortality. Thorax.

[35] Montalescot G., Sechtem U., Achenbach S., Andreotti F., Arden C., Budaj A., Bugiardini R., Crea F., Cuisset T., Di Mario C. (2013). 2013 ESC guidelines on the management of stable coronary artery disease: The Task Force on the management of stable coronary artery disease of the European Society of Cardiology. Eur. Heart J..

[36] Almagro P., Lapuente A., Pareja J., Yun S., Garcia M.E., Padilla F., Heredia J.L., De la Sierra A., Soriano J.B. (2015). Underdiagnosis and prognosis of chronic obstructive pulmonary disease after percutaneous coronary intervention: A prospective study. Int. J. Chronic Obstr. Pulm. Dis..

[37] Brekke P.H., Omland T., Smith P., Soyseth V. (2008). Underdiagnosis of myocardial infarction in COPD—Cardiac Infarction Injury Score (CIIS) in patients hospitalised for COPD exacerbation. Respir. Med..

[38] Andell P., Erlinge D., Smith J.G., Sundstrom J., Lindahl B., James S., Koul S. (2015). Beta-blocker use and mortality in COPD patients after myocardial infarction: A Swedish nationwide observational study. J. Am. Heart Assoc..

[39] Goldberger J.J., Bonow R.O., Cuffe M., Dyer A., Rosenberg Y., O’Rourke R., Shah P.K., Smith S.C., Investigators P.-M. (2010). β-Blocker use following myocardial infarction: Low prevalence of evidence-based dosing. Am. Heart J..

[40] Salpeter S.R., Ormiston T.M., Salpeter E.E. (2002). Cardioselective β-blockers in patients with reactive airway disease: A meta-analysis. Ann. Intern. Med..

[41] Rutten F.H., Zuithoff N.P., Hak E., Grobbee D.E., Hoes A.W. (2010). Beta-blockers may reduce mortality and risk of exacerbations in patients with chronic obstructive pulmonary disease. Arch. Intern. Med..

[42] Ferrari R., Camici P.G., Crea F., Danchin N., Fox K., Maggioni A.P., Manolis A.J., Marzilli M., Rosano G.M.C., Lopez-Sendon J.L. (2018). Expert consensus document: A ‘diamond’ approach to personalized treatment of angina. Nat. Rev. Cardiol..

[43] Mascitelli L., Pezzetta F., Goldstein M.R. (2008). Inhibition of the renin-angiotensin system in severe COPD. Eur. Respir. J..

[44] Shrikrishna D., Astin R., Kemp P.R., Hopkinson N.S. (2012). Renin-angiotensin system blockade: A novel therapeutic approach in chronic obstructive pulmonary disease. Clin. Sci..

[45] Packard K.A., Wurdeman R.L., Arouni A.J. (2002). ACE inhibitor-induced bronchial reactivity in patients with respiratory dysfunction. Ann. Pharmacother..

[46] Tanaka H., Teramoto S., Oashi K., Saikai T., Tanaka S., Suzuki K., Hashimoto M., Abe S. (2001). Effects of candesartan on cough and bronchial hyperresponsiveness in mildly to moderately hypertensive patients with symptomatic asthma. Circulation.

[47] Caldeira D., Pinto F.J., Ferreira J.J. (2014). Dyspnea and reversibility profile of P2Y(1)(2) antagonists: Systematic review of new antiplatelet drugs. Am. J. Cardiovasc. Drugs.

[48] Gershon A., Croxford R., Calzavara A., To T., Stanbrook M.B., Upshur R., Stukel T.A. (2013). Cardiovascular safety of inhaled long-acting bronchodilators in individuals with chronic obstructive pulmonary disease. JAMA Intern. Med..

[49] Salpeter S.R., Ormiston T.M., Salpeter E.E. (2004). Cardiovascular effects of beta-agonists in patients with asthma and COPD: A meta-analysis. Chest.

[50] Rottenkolber M., Rottenkolber D., Fischer R., Ibanez L., Fortuny J., Ballarin E., Sabate M., Ferrer P., Thurmann P., Hasford J. (2014). Inhaled beta-2-agonists/muscarinic antagonists and acute myocardial infarction in COPD patients. Respir. Med..

[51] Lee C.H., Choi S., Jang E.J., Yang H.M., Il Yoon H., Kim Y.J., Kim J., Yim J.J., Kim D.K. (2017). Inhaled bronchodilators and acute myocardial infarction: A nested case-control study. Sci. Rep..

[52] Rogliani P., Matera M.G., Ora J., Cazzola M., Calzetta L. (2017). The impact of dual bronchodilation on cardiovascular serious adverse events and mortality in COPD: A quantitative synthesis. Int. J. Chronic Obstr. Pulm. Dis..

[53] Leavitt B.J., Ross C.S., Spence B., Surgenor S.D., Olmstead E.M., Clough R.A., Charlesworth D.C., Kramer R.S., O’Connor G.T. (2006). Northern New England Cardiovascular Disease Study Group. Long-term survival of patients with chronic obstructive pulmonary disease undergoing coronary artery bypass surgery. Circulation.

[54] Selvaraj C.L., Gurm H.S., Gupta R., Ellis S.G., Bhatt D.L. (2005). Chronic obstructive pulmonary disease as a predictor of mortality in patients undergoing percutaneous coronary intervention. Am. J. Cardiol..

[55] Enriquez J.R., Parikh S.V., Selzer F., Jacobs A.K., Marroquin O., Mulukutla S., Srinivas V., Holper E.M. (2011). Increased adverse events after percutaneous coronary intervention in patients with COPD: Insights from the National Heart, Lung, and Blood Institute dynamic registry. Chest.

[56] Windecker S., Kolh P., Alfonso F., Collet J.P., Cremer J., Falk V., Filippatos G., Hamm C., Head S.J., Juni P. (2014). 2014 ESC/EACTS Guidelines on myocardial revascularization: The Task Force on Myocardial Revascularization of the European Society of Cardiology (ESC) and the European Association for Cardio-Thoracic Surgery (EACTS)Developed with the special contribution of the European Association of Percutaneous Cardiovascular Interventions (EAPCI). Eur. Heart J..

[57] Huerta C., Lanes S.F., Garcia Rodriguez L.A. (2005). Respiratory medications and the risk of cardiac arrhythmias. Epidemiology.

[58] Van der Hooft C.S., Heeringa J., Brusselle G.G., Hofman A., Witteman J.C., Kingma J.H., Sturkenboom M.C., Stricker B.H. (2006). Corticosteroids and the risk of atrial fibrillation. Arch. Intern. Med..

[59] Brook R.D., Anderson J.A., Calverley P.M., Celli B.R., Crim C., Denvir M.A., Magder S., Martinez F.J., Rajagopalan S., Vestbo J. (2017). Cardiovascular outcomes with an inhaled beta2-agonist/corticosteroid in patients with COPD at high cardiovascular risk. Heart.

[60] Salpeter S.R. (2009). Do inhaled anticholinergics increase or decrease the risk of major cardiovascular events? A synthesis of the available evidence. Drugs.

[61] Sessler C.N., Cohen M.D. (1990). Cardiac arrhythmias during theophylline toxicity. A prospective continuous electrocardiographic study. Chest.

[62] Liang M., Puri A., Devlin G. (2009). Heart rate and cardiovascular disease: An alternative to Beta blockers. Cardiol. Res. Pract..

[63] Camus P., Martin W.J., Rosenow E.C. (2004). Amiodarone pulmonary toxicity. Clin. Chest Med..

[64] Rutten F.H., Cramer M.J., Grobbee D.E., Sachs A.P., Kirkels J.H., Lammers J.W., Hoes A.W. (2005). Unrecognized heart failure in elderly patients with stable chronic obstructive pulmonary disease. Eur. Heart J..

[65] Macchia A., Rodriguez Moncalvo J.J., Kleinert M., Comignani P.D., Gimeno G., Arakaki D., Laffaye N., Fuselli J.J., Massolin H.P., Gambarte J. (2012). Unrecognised ventricular dysfunction in COPD. Eur. Respir. J..

[66] Rusinaru D., Saaidi I., Godard S., Mahjoub H., Battle C., Tribouilloy C. (2008). Impact of chronic obstructive pulmonary disease on long-term outcome of patients hospitalized for heart failure. Am. J. Cardiol..

[67] Brenner S., Guder G., Berliner D., Deubner N., Frohlich K., Ertl G., Jany B., Angermann C.E., Stork S. (2013). Airway obstruction in systolic heart failure—COPD or congestion?. Int. J. Cardiol..

[68] Le Jemtel T.H., Padeletti M., Jelic S. (2007). Diagnostic and therapeutic challenges in patients with coexistent chronic obstructive pulmonary disease and chronic heart failure. J. Am. Coll. Cardiol..

[69] Flessas N., Alexanian I., Parissis J., Kremastinos D., Lekakis J., Filippatos G. (2014). Plasma activity of B-type natriuretic peptide in patients with biventricular heart failure versus those with right heart failure due to chronic obstructive pulmonary disease. J. Cardiovasc. Med..

[70] Hawkins N.M., Virani S., Ceconi C. (2013). Heart failure and chronic obstructive pulmonary disease: The challenges facing physicians and health services. Eur. Heart J..

[71] Caruana L., Petrie M.C., Davie A.P., McMurray J.J. (2000). Do patients with suspected heart failure and preserved left ventricular systolic function suffer from “diastolic heart failure” or from misdiagnosis? A prospective descriptive study. BMJ.

[72] Mühle A., Obst A., Winkler J., Ewert R. (2015). Cardiopulmonary Exercise Testing in Chronic Obstructive Pulmonary Disease (COPD)—Breath-functional Characterization and Disease Severity Assessment. Pneumologie.

[73] Rutten F.H., Cramer M.J., Zuithoff N.P., Lammers J.W., Verweij W., Grobbee D.E., Hoes A.W. (2007). Comparison of B-type natriuretic peptide assays for identifying heart failure in stable elderly patients with a clinical diagnosis of chronic obstructive pulmonary disease. Eur. J. Heart Fail..

[74] Lipworth B., Skinner D., Devereux G., Thomas V., Jie J.L., Martin J., Carter V., Price D.B. (2016). Underuse of beta-blockers in heart failure and chronic obstructive pulmonary disease. Heart.

[75] Ponikowski P., Voors A.A., Anker S.D., Bueno H., Cleland J.G., Coats A.J., Falk V., Gonzalez-Juanatey J.R., Harjola V.P., Jankowska E.A. (2016). 2016 ESC Guidelines for the diagnosis and treatment of acute and chronic heart failure: The Task Force for the diagnosis and treatment of acute and chronic heart failure of the European Society of Cardiology (ESC) Developed with the special contribution of the Heart Failure Association (HFA) of the ESC. Eur. Heart J..

[76] Lainscak M., Podbregar M., Kovacic D., Rozman J., von Haehling S. (2011). Differences between bisoprolol and carvedilol in patients with chronic heart failure and chronic obstructive pulmonary disease: A randomized trial. Respir. Med..

[77] Hohlfeld J.M., Vogel-Claussen J., Biller H., Berliner D., Berschneider K., Tillmann H.C., Hiltl S., Bauersachs J., Welte T. (2018). Effect of lung deflation with indacaterol plus glycopyrronium on ventricular filling in patients with hyperinflation and COPD (CLAIM): A double-blind, randomised, crossover, placebo-controlled, single-centre trial. Lancet Respir. Med..

[78] Wheaton A.G., Ford E.S., Cunningham T.J., Croft J.B. (2015). Chronic obstructive pulmonary disease, hospital visits, and comorbidities: National Survey of Residential Care Facilities, 2010. J. Aging Health.

[79] Mills N.L., Miller J.J., Anand A., Robinson S.D., Frazer G.A., Anderson D., Breen L., Wilkinson I.B., McEniery C.M., Donaldson K. (2008). Increased arterial stiffness in patients with chronic obstructive pulmonary disease: A mechanism for increased cardiovascular risk. Thorax.

[80] De Luca N., Asmar R.G., London G.M., O’Rourke M.F., Safar M.E. (2004). Selective reduction of cardiac mass and central blood pressure on low-dose combination perindopril/indapamide in hypertensive subjects. J. Hypertens..

[81] Roman M.J., Devereux R.B., Kizer J.R., Lee E.T., Galloway J.M., Ali T., Umans J.G., Howard B.V. (2007). Central pressure more strongly relates to vascular disease and outcome than does brachial pressure: The Strong Heart Study. Hypertension.

[82] Wang K.L., Cheng H.M., Chuang S.Y., Spurgeon H.A., Ting C.T., Lakatta E.G., Yin F.C., Chou P., Chen C.H. (2009). Central or peripheral systolic or pulse pressure: Which best relates to target organs and future mortality?. J. Hypertens..

[83] Pini R., Cavallini M.C., Palmieri V., Marchionni N., Di Bari M., Devereux R.B., Masotti G., Roman M.J. (2008). Central but not brachial blood pressure predicts cardiovascular events in an unselected geriatric population: The ICARe Dicomano Study. J. Am. Coll. Cardiol..

[84] Protogerou A.D., Stergiou G.S., Vlachopoulos C., Blacher J., Achimastos A. (2009). The effect of antihypertensive drugs on central blood pressure beyond peripheral blood pressure. Part II: Evidence for specific class-effects of antihypertensive drugs on pressure amplification. Curr. Pharm. Des..

[85] Manisty C.H., Hughes A.D. (2013). Meta-analysis of the comparative effects of different classes of antihypertensive agents on brachial and central systolic blood pressure, and augmentation index. Br. J. Clin. Pharmacol..

[86] Boutouyrie P., Achouba A., Trunet P., Laurent S. (2010). Amlodipine-valsartan combination decreases central systolic blood pressure more effectively than the amlodipine-atenolol combination: The EXPLOR study. Hypertension.

[87] Williams B., Lacy P.S., Thom S.M., Cruickshank K., Stanton A., Collier D., Hughes A.D., Thurston H., O’Rourke M. (2006). Differential impact of blood pressure-lowering drugs on central aortic pressure and clinical outcomes: Principal results of the Conduit Artery Function Evaluation (CAFE) study. Circulation.

[88] Chandy D., Aronow W.S., Banach M. (2013). Current perspectives on treatment of hypertensive patients with chronic obstructive pulmonary disease. Integr. Blood Press. Control.

[89] Cheng H.M., Lang D., Tufanaru C., Pearson A. (2013). Measurement accuracy of non-invasively obtained central blood pressure by applanation tonometry: A systematic review and meta-analysis. Int. J. Cardiol..

[90] Papaioannou T.G., Karageorgopoulou T.D., Sergentanis T.N., Protogerou A.D., Psaltopoulou T., Sharman J.E., Weber T., Blacher J., Daskalopoulou S.S., Wassertheurer S. (2016). Accuracy of commercial devices and methods for noninvasive estimation of aortic systolic blood pressure a systematic review and meta-analysis of invasive validation studies. J. Hypertens..

[91] Schumacher G., Kaden J.J., Trinkmann F. (2017). Multiple coupled resonances in the human vascular tree—Refining the Westerhof model of the arterial system. J. Appl. Physiol..

[92] Seeger W., Adir Y., Barbera J.A., Champion H., Coghlan J.G., Cottin V., De Marco T., Galie N., Ghio S., Gibbs S. (2013). Pulmonary hypertension in chronic lung diseases. J. Am. Coll. Cardiol..

[93] Guazzi M., Borlaug B.A. (2012). Pulmonary hypertension due to left heart disease. Circulation.

[94] Terzano C., Romani S., Conti V., Paone G., Oriolo F., Vitarelli A. (2014). Atrial fibrillation in the acute, hypercapnic exacerbations of COPD. Eur. Rev. Med. Pharmacol. Sci..

[95] Buch P., Friberg J., Scharling H., Lange P., Prescott E. (2003). Reduced lung function and risk of atrial fibrillation in the Copenhagen City Heart Study. Eur. Respir. J..

[96] Kirchhof P., Benussi S., Kotecha D., Ahlsson A., Atar D., Casadei B., Castella M., Diener H.C., Heidbuchel H., Hendriks J. (2016). 2016 ESC Guidelines for the management of atrial fibrillation developed in collaboration with EACTS. Eur. Heart J..

[97] Gu J., Liu X., Tan H., Zhou L., Jiang W., Wang Y., Liu Y., Gu J. (2013). Impact of chronic obstructive pulmonary disease on procedural outcomes and quality of life in patients with atrial fibrillation undergoing catheter ablation. J. Cardiovasc. Electrophysiol..

[98] Pisters R., Nieuwlaat R., Prins M.H., Le Heuzey J.Y., Maggioni A.P., Camm A.J., Crijns H.J. (2012). Clinical correlates of immediate success and outcome at 1-year follow-up of real-world cardioversion of atrial fibrillation: The Euro Heart Survey. Europace.

[99] Roh S.Y., Choi J.I., Lee J.Y., Kwak J.J., Park J.S., Kim J.B., Lim H.E., Kim Y.H. (2011). Catheter ablation of atrial fibrillation in patients with chronic lung disease. Circulation.

[100] McCord J., Borzak S. (1998). Multifocal atrial tachycardia. Chest.

[101] Lahousse L., Niemeijer M.N., van den Berg M.E., Rijnbeek P.R., Joos G.F., Hofman A., Franco O.H., Deckers J.W., Eijgelsheim M., Stricker B.H. (2015). Chronic obstructive pulmonary disease and sudden cardiac death: The Rotterdam study. Eur. Heart J..

[102] Priori S.G., Blomstrom-Lundqvist C., Mazzanti A., Blom N., Borggrefe M., Camm J., Elliott P.M., Fitzsimons D., Hatala R., Hindricks G. (2015). 2015 ESC Guidelines for the management of patients with ventricular arrhythmias and the prevention of sudden cardiac death: The Task Force for the Management of Patients with Ventricular Arrhythmias and the Prevention of Sudden Cardiac Death of the European Society of Cardiology (ESC) Endorsed by: Association for European Paediatric and Congenital Cardiology (AEPC). Europace.

